# Flow Diversion for Cerebral Aneurysms: A Decade-Long Experience with Improved Outcomes and Predictors of Success

**DOI:** 10.3390/brainsci14080847

**Published:** 2024-08-22

**Authors:** Tae Keun Jee, Je Young Yeon, Keon Ha Kim, Jong-Soo Kim, Pyoung Jeon

**Affiliations:** 1 Department of Neurosurgery, Samsung Medical Center, Sungkyunkwan University School of Medicine, Seoul 06351, Republic of Korea; taekeun.jee@samsung.com (T.K.J.); yeonjay.youn@samsung.com (J.Y.Y.);; 2 Department of Radiology, Samsung Medical Center, Sungkyunkwan University School of Medicine, Seoul 06351, Republic of Korea

**Keywords:** flow diversion, cerebral aneurysm, aneurysm occlusion, long-term outcomes, safety outcomes

## Abstract

Background: Flow diversion has significantly improved the management of cerebral aneurysms. Technological advancements and increased clinical experience over the past decade have led to better outcomes and fewer complications. This study provides updated results and examines the factors that influence the success of flow diversion. Methods: We reviewed records of 115 patients with 121 intracranial aneurysms treated from July 2014 to August 2023. All patients had unruptured aneurysms in the anterior and posterior circulation. Results: Complete aneurysm occlusion was achieved in 72.7% of cases, with a complication rate of 9.1%. Significant predictors of complete occlusion included aneurysm diameter (OR = 0.89, 95% CI 0.82–0.97, *p* = 0.009) and the presence of incorporated branches (OR = 0.22, 95% CI 0.08–0.59, *p* = 0.003). Cox analysis identified neck diameter (HR = 0.92, 95% CI 0.87–0.98, *p* = 0.009) and incorporated branch (HR = 0.40, 95% CI 0.24–0.69, *p* = 0.001) as significant for occlusion. Multivariable analysis identified aneurysm diameter (OR = 1.21, 95% CI 1.09–1.37, *p* = 0.001) as significant for safety outcomes. Improved outcomes were observed in recent treatments, with higher occlusion rates (79.7% vs. 61.7%, *p* = 0.050) and lower complication rates (4.1% vs. 14.9%, *p* = 0.011). Conclusions: Enhanced technical proficiency, better devices, and refined patient selection have significantly improved the efficacy and safety of flow diversion for cerebral aneurysms. Identifying significant predictors for treatment success and safety outcomes can inform clinical practice, aiding in patient selection.

## 1. Introduction

Flow diversion stents have revolutionized the treatment of cerebral aneurysms, particularly those that are difficult to manage with traditional methods [1,2]. These stents redirect blood flow away from the aneurysm, promoting thrombosis and eventual occlusion. Numerous studies have been conducted on flow diversion, with early research highlighting its promising outcomes and subsequent studies providing evidence on when flow diversion is more advantageous [3,4,5,6]. Understanding the specific characteristics and outcomes associated with flow diversion is essential for improving the overall treatment results. When unfavorable outcomes occur after flow diversion, subsequent treatment options are significantly limited, often constrained to either deploying additional flow diverters or performing parent artery occlusion with or without bypass surgery [6,7]. In 2021, we reported our experience on the use of flow diversion stents for large or giant aneurysms [5], demonstrating a relatively low complete occlusion rate (57.1%) and a high complication rate (17.1%) compared to other studies [8,9,10,11,12]. Since then, we have treated a larger patient population and extended the follow-up period, resulting in a significant accumulation of data. This study aims to present updated results and analyze the factors contributing to treatment outcomes after flow diversion. Additionally, with increased experience, we have observed improvements in treatment outcomes, including better aneurysm occlusion rates and reduced complications. By sharing our decade-long experience, we aim to contribute to the optimization of flow diversion stent protocols and improve treatment efficacy.

## 2. Methods

This study received approval from the institutional review board (IRB File No. 2021-01-176-001) of our institution. We conducted a review of the medical records of patients who underwent flow diversion for intracranial aneurysms at our facility from July 2014 to August 2023. The decision for flow diversion treatment was made based on multidisciplinary team discussions involving neurosurgeons and interventional neuroradiologists, as well as patient preferences. The study population included individuals with unruptured aneurysms in both the anterior and posterior circulation. Patients who were lost to follow-up and had less than 12 months of angiographic follow-up before achieving complete aneurysm occlusion without adverse events were excluded from the analysis, whereas patients who experienced any type of safety outcome were included in the analysis despite having less than 12 months of angiographic follow-up. Clinical data such as age, sex, medical history, and results of platelet reactivity testing were obtained from the medical records of the patients included in the study. The diameter and neck size of the aneurysms were assessed using three-dimensional digital subtraction angiography (DSA), with the maximum value of each dimension recorded. For aneurysms containing luminal thrombi, the diameter was measured as the outer-to-outer diameter on pre-treatment magnetic resonance imaging (MRI).

### 2.1. Interventions and Follow-Up

All patients received premedication with a dual antiplatelet regimen consisting of either 100 mg aspirin and 75 mg clopidogrel for 5 to 14 days or a loading dose of 300 mg aspirin and clopidogrel for 1 to 2 days prior to the flow diversion procedure. Platelet reactivity was assessed using the VerifyNow Assay (Accumetrics Inc., San Diego, CA, USA). For patients exhibiting clopidogrel hyporesponsiveness (P2Y12 reaction units > 230), ticlopidine (250 mg) was administered twice daily as an alternative. The dual antiplatelet therapy continued for 6 months post-procedure, followed by a single antiplatelet agent for an additional 6 months.

Three types of commercial flow diversion systems were used: the Pipeline Embolization Device (Flex and Flex with Shield technology, Medtronic, Irvine, CA, USA), the Derivo Embolization Device (Acandis GmbH & Co, KG, Pforzheim, Germany), and the Surpass Flow Diverter (Streamline and Evolve, Stryker Neurovascular, CA, USA). The choice of device was based on the operator’s preference. Post-procedural MRI was performed within 5 days of the procedure. Clinical and radiological follow-ups included MRI and/or CT angiography at 6, 12, 18, and 24 months after the procedure and annually thereafter. Follow-up assessments were conducted at the discretion of the attending physician.

### 2.2. Outcome Measures

Complete aneurysm occlusion was defined as the angiographic occlusion of the target aneurysm at the end of follow-up without significant (>50%) parent artery stenosis, major adverse events, or the need for additional treatment. Safety outcomes included hemorrhagic stroke, major ischemic stroke (defined as an increase of ≥4 points on the National Institutes of Health Stroke Scale), partial or complete stent thrombosis, and all-cause mortality. Significant enlargement of the target aneurysm and unfavorable functional outcomes were also assessed. As previously described in our study, significant enlargement of the target aneurysm was defined as a follow-up aneurysm volume exceeding 125% of the initial aneurysm volume [7]. The aneurysmal diameter was measured on MRI using the outer-to-outer diameter. Functional outcomes were assessed at an outpatient clinic, with unfavorable functional outcomes defined as a modified Rankin Scale score of 3–6 at the last clinical follow-up. Additionally, we compared the baseline characteristics, treatment details, and treatment outcomes between the early group and the recent group, categorized based on the time of the previous study publication.

### 2.3. Statistical Analyses

Statistical analyses were performed using SPSS Statistics version 25.0 (IBM Corporation, Armonk, NY, USA) and R version 3.4.2 (R Foundation for Statistical Computing, Vienna, Austria). A *p* value of <0.05 was considered indicative of statistical significance, with all *p* values based on two-sided tests. Differences in categorical variables between groups were analyzed using the chi-square test with continuity correction, while continuous variables were compared using the T-test. To determine associations between clinical variables and complete aneurysm occlusion, univariate binary logistic regression analysis was conducted. A multivariate logistic regression model, with a significance level set at 0.20, was then used to identify independent predictors. Odds ratios (ORs) and 95% confidence intervals (CIs) were calculated for the identified risk variables. A Cox proportional hazard analysis was performed to assess time-dependent factors influencing complete aneurysm occlusion after flow diversion, also with a significance level set at 0.20. Hazards ratios (HRs) and 95% confidence intervals (CIs) were calculated for the identified risk variables.

## 3. Results

During the study period, 128 patients with 134 intracranial aneurysms underwent flow diversion treatment. However, 13 patients with 13 intracranial aneurysms were excluded for the following reasons: incomplete angiographic follow-up (*n* = 11) or intracranial artery dissection without aneurysm formation (*n* = 2). Consequently, the final analysis included 115 patients with 121 aneurysms.

Table 1 summarizes the clinicoradiological findings of 121 aneurysms; the patients had a mean age of 59.4 ± 12.9 years and 82 (67.8%) were female. Regarding previous treatments, the majority had no prior interventions (113 patients, 93.4%), while two patients (1.7%) had undergone clipping. Additionally, one patient each had distal occlusion and bypass (0.8%) and multiple stents (0.8%). Coil embolization had been performed in four patients (3.3%). The majority of aneurysms were located in the anterior circulation (85 patients, 70.2%). The mean aneurysm diameter was 15.2 ± 7.0 mm, with sizes categorized as <10 mm in 24 patients (19.8%), 10–25 mm in 82 patients (67.8%), and ≥25 mm in 15 patients (12.4%). The mean neck diameter was 8.9 ± 4.8 mm, and 33 patients (27.3%) had an incorporated branch preventing aneurysm isolation after flow diversion.

The devices used were the Surpass Evolve in 67 patients (55.4%), Surpass Streamline in 31 patients (25.6%), Pipeline Embolization Device in 22 patients (18.2%), and Derivo Embolization Device in 1 patient (0.8%), with additional coils used in 3 patients (2.5%). The number of flow diverters used was 1 in 116 patients (95.9%) and 2 in 5 patients (4.1%). Balloon angioplasty was performed in 40 patients (33.1%), and the mean procedure time was 91.1 ± 44.9 min. The mean angiographic follow-up was 26.0 ± 19.0 months, and the mean clinical follow-up was 29.5 ± 22.2 months.

Complete aneurysm occlusion was achieved in 88 patients (72.7%). The mean duration from flow diversion to aneurysm occlusion was 14.7 ± 15.6 months. Safety outcomes were noted in 11 patients (9.1%), including hemorrhagic events in 4 patients (3.3%) with 2 cases of delayed RIPH (1.7%) and 2 cases of delayed rupture with subarachnoid hemorrhage (SAH) (1.6%). Major infarction occurred in four patients (3.3%), with downstream embolic infarction in one patient (0.8%) and covered perforator territory infarction in one patient (0.8%). Stent thrombosis with infarction was observed in two patients (1.7%). Stent thrombosis without infarction was also noted in two patients (1.7%). There was one case of sudden death from an unknown cause (0.8%). Unfavorable functional outcomes were reported in five patients (4.1%); aneurysm enlargement was seen in 14 patients (11.6%). The treatment outcomes are summarized in Table 2.

**Figure 1:**
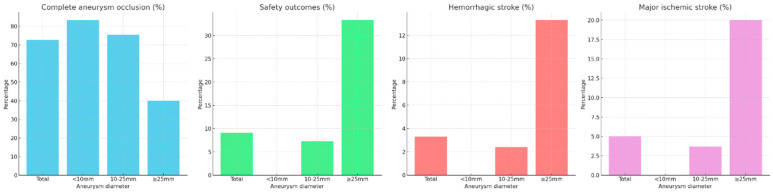
Outcomes of flow diversion treatment stratified by aneurysm diameter.

Table 3 presents the results of the logistic regression analysis for complete aneurysm occlusion, safety outcomes, and all stroke (hemorrhagic and major ischemic stroke). In the univariate analysis for aneurysm occlusion, significant factors included non-saccular type (*p* = 0.048, OR = 0.49, 95% CI 0.19–0.99), aneurysm diameter (*p* < 0.001, OR = 0.89, 95% CI 0.83–0.95), neck diameter (*p* = 0.004, OR = 0.85, 95% CI 0.77–0.94), and incorporated branch (*p* = 0.002, OR = 0.25, 95% CI 0.11–0.60). Multivariate analysis showed aneurysm diameter (*p* = 0.009, OR = 0.89, 95% CI 0.82–0.97) and incorporated branch (*p* = 0.003, OR = 0.22, 95% CI 0.08–0.59) remained significant. For safety outcomes, univariate analysis identified aneurysm diameter (*p* < 0.001, OR = 1.20, 95% CI 1.09–1.34) as a significant factor. Multivariate analysis confirmed aneurysm diameter (*p* = 0.001, OR = 1.21, 95% CI 1.09–1.37) as significant. For hemorrhagic and major ischemic stroke, univariate analysis indicated aneurysm diameter (*p* = 0.003, OR = 1.18, 95% CI 1.06–1.33) as significant. Multivariate analysis validated aneurysm diameter (*p* = 0.003, OR = 1.18, 95% CI 1.06–1.33) as a significant factor. Aneurysm diameter is the most important factor, significantly influencing all treatment outcomes, including complete aneurysm occlusion, safety outcomes, and hemorrhagic and ischemic strokes. Figure 1 shows the percentage of each treatment outcome based on the aneurysm diameter, highlighting that larger aneurysms have lower complete aneurysm occlusion rates and higher complication rates.

**Figure 2:**
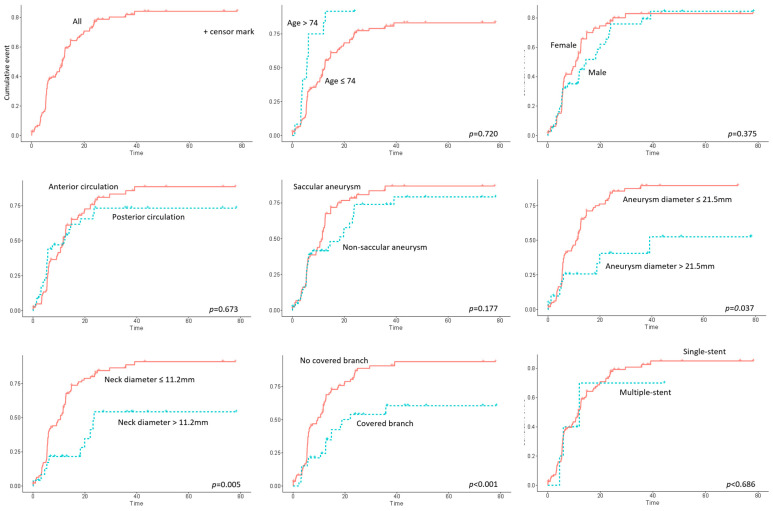
Cumulative event analysis for aneurysm occlusion stratified by various clinical and anatomical factors.

The results of the Cox proportional hazard analysis are summarized in Table 4 and Figure 2. Univariate analysis identified aneurysm diameter (*p* = 0.037, HR = 0.97, 95% CI 0.94–1.00), neck diameter (*p* = 0.006, HR = 0.93, 95% CI 0.88–0.98), and incorporated branch (*p* = 0.001, HR = 0.39, 95% CI 0.23–0.66) as significant factors. Multivariate analysis confirmed that neck diameter (*p* = 0.009, HR = 0.92, 95% CI 0.87–0.98) and incorporated branch (*p* = 0.001, HR = 0.40, 95% CI 0.24–0.69) remained significant predictors for aneurysm occlusion.

Table 5 presents a comparison of baseline characteristics, treatment details, and clinical outcomes between the early group (*n* = 47) and the recent group (*n* = 74). The recent group had a significantly higher proportion of female patients (79.7% vs. 48.9%, *p* = 0.001). Non-saccular aneurysms were more prevalent in the early group (53.2% vs. 27.0%, *p* = 0.007). The early group also had larger mean aneurysm diameters (19.3 ± 6.2 mm vs. 12.6 ± 6.3 mm, *p* < 0.001) and neck diameters (10.3 ± 5.3 mm vs. 7.9 ± 4.1 mm, *p* = 0.005). Balloon angioplasty was performed more often in the early group (46.8% vs. 24.3%, *p* = 0.018), and their procedures took longer on average (113.4 ± 52.8 min vs. 77.0 ± 32.2 min, *p* < 0.001). The recent group had a higher rate of aneurysm occlusion (79.7% vs. 61.7%, *p* = 0.050) and a lower incidence of aneurysm enlargement (2.7% vs. 25.5%, *p* < 0.001). Additionally, the early group experienced higher rates of all strokes (14.9% vs. 1.4%, *p* = 0.011) and hemorrhagic stroke (8.5% vs. 0%, *p* = 0.042).

## 4. Discussion

Our study provides a detailed analysis of flow diversion treatment outcomes for intracranial aneurysms, focusing on factors affecting complete aneurysm occlusion and safety outcomes. The complete aneurysm occlusion rate of 72.7% was consistent with that observed in previous large cohort studies [8,9,10,11,12,13]. The safety outcomes, with a complication rate of 9.1%, reflect the inherent risks associated with the procedure but also highlight areas for potential improvement in patient management and pre-procedural planning. Notably, the recent group exhibited a lower complication rate of 4.1%, suggesting that careful case selection and appropriate procedural techniques can significantly enhance patient safety. In a meta-analysis encompassing 29 studies and 1654 aneurysms, Brinjikji et al. reported that subarachnoid hemorrhage from a delayed aneurysm rupture and ischemic stroke occurred in 4% and 6% of patients, respectively, following flow diversion treatment [3]. These complications were notably more frequent in patients with large and giant aneurysms. Another recent meta-analysis indicates that unruptured non-saccular aneurysms in the posterior or distal anterior circulation can be effectively treated with flow diversion, despite notable complication rates (15% ischemic events, 8% morbidity). Larger aneurysms (>10 mm) are associated with higher risks of adverse events [4]. In our study, the mean aneurysm diameter was 15.2 ± 7.0 mm, with 62.8% being non-saccular aneurysms and 38% located in the posterior or distal anterior circulation. Given this composition, our study results are favorable and reaffirm the efficacy of flow diversion for these complex aneurysms.

Technological advancements in flow diversion devices and increased experience in high-volume centers have shown that treatment outcomes are significantly influenced by case selection rather than the stent deployment technique itself [14,15]. From this standpoint, case selection is the most critical factor for treatment outcomes and understanding predictors of favorable outcomes is crucial. Previous studies have identified several predictors, consistently highlighting aneurysm size, the presence of an incorporated vessel, and the location of the aneurysm, particularly if it is distal or posterior [3,4,5,16,17,18]. In the present study, logistic regression analysis revealed significant predictors for aneurysm occlusion, including aneurysm diameter and the presence of an incorporated vessel. Aneurysm diameter was also a significant predictor for safety outcomes and stroke events. The Cox proportional hazard analysis provided additional insights into the time-dependent factors influencing aneurysm occlusion. Neck diameter and the presence of an incorporated branch were significant predictors. These results further emphasize the importance of aneurysm morphology in determining treatment success.

### 4.1. Comparison between Early and Recent Treatment Groups

Our comparison between the early and recent treatment groups revealed significant improvements in outcomes over time, driven by several key factors. As our experience with flow diversion procedures grew, so did our technical proficiency. This is clearly demonstrated by the decreased procedure time in the recent group, averaging 77.0 min compared to 113.4 min in the early group. This reduction in procedure time underscores the increased efficiency and skill development we achieved with continued practice and familiarity with the technique. Another major contributor to the improved outcomes is the advancement in flow diverter devices. The newer devices we adopted over time offer enhanced deliverability and safety features that have significantly bolstered the effectiveness of the treatment. These technological advancements have allowed us to perform the procedures with greater precision and reliability, thereby improving patient outcomes [14,19]. However, the most significant factor contributing to the better results in the recent group is our refined patient selection process. Initially, our selection criteria were broader and our understanding of the factors leading to unfavorable outcomes was less developed. Over time, insights gained from our earlier studies, which identified critical factors such as aneurysm diameter, incorporated branches, and parent vessel angle, allowed us to hone our criteria. This refinement has enabled us to select patients who are more likely to benefit from flow diversion, thereby enhancing overall outcomes and minimizing complications.

### 4.2. Implications for Clinical Practice

The identification of significant predictors for both treatment success and safety outcomes can inform patient selection criteria, helping clinicians identify those who are most likely to benefit from flow diversion. Flow diversion is a highly valuable treatment option for cerebral aneurysms; however, it is not universally applicable to all complex aneurysm cases. When flow diversion fails, particularly in cases of aneurysm enlargement, delayed rupture, or clinical deterioration, the subsequent treatment options are limited. These often include additional flow diverter deployment or parent artery occlusion with or without bypass surgery. Therefore, it is crucial to consider alternative conventional treatments when unfavorable outcomes are anticipated with flow diversion [20]. However, despite careful consideration and meticulous inspection, finding a promising treatment option for complex aneurysms is difficult and sometimes even impossible. According to the results of our analysis, large or giant aneurysms with wide necks and incorporated branches present significant concerns regarding safety outcomes and treatment efficacy after flow diversion. Although the non-saccular type did not demonstrate statistical significance in the present study, it could be a potential factor associated with unfavorable outcomes. This type may enhance the impact of other poor outcome factors, such as large size and incorporated branches, particularly when they coexist. However, the present study had limitations in fully elucidating this relationship. When technically feasible, our center has increasingly opted for surgical treatment options for such aneurysms compared to the early stage of flow diversion. Since most of these aneurysms are unclippable, surgical options often involve parent artery trapping with bypass or hybrid techniques. We plan to address this topic in a future report.

### 4.3. Limitations

This study has several limitations. Conducted at a single center, its findings may not be generalizable to other settings. The retrospective design introduces potential biases due to incomplete or missing data and limits the ability to establish causality. The follow-up period, although long, might still miss late complications or recurrences.

## 5. Conclusions

In conclusion, the combination of increased technical proficiency, advancements in flow diverter devices, and refined patient selection criteria has led to significant improvements in the success of flow diversion treatments over time. These enhancements have collectively contributed to better aneurysm occlusion rates and reduced complication rates, demonstrating the value of experience, technological innovation, and strategic patient selection in optimizing treatment outcomes.

## Figures and Tables

**Table 1:** Clinicoradiological findings of the total 121 aneurysms.

Age (year)	59.4 ± 12.9
Female	82 (67.8%)
Previous treatment	
-No	113 (93.4%)
-Clipping	2 (1.7%)
-Distal occlusion, Bypass	1 (0.8%)
-Multiple stents	1 (0.8%)
-Coil embolization	4 (3.3%)
Anterior circulation	85 (70.2%)
Location	
-ICA	75 (62.0%)
-ACA	4 (3.3%)
-MCA	6 (5.0%)
-PCA	1 (0.8%)
-BA	6 (5.0%)
-VA	29 (24.0%)
Non-saccular type	76 (62.8%)
Aneurysm diameter (mm)	15.2 ± 7.0
-<10	24 (19.8%)
-10–25	82 (67.8%)
-≥25	15 (12.4%)
Neck diameter (mm)	8.9 ± 4.8
Incorporated branch	33 (27.3%)
Pre-treatment mRS	
-0	112 (92.6%)
-1	8 (6.6%)
-2	1 (0.8%)
Pre-treatment DAPT	
-On DAPT	4 (3.3%)
-Loading dose	8 (6.6%)
-Scheduled	109 (90.1%)
Pre-treatment DAPT regimen	
-Aspirin + Cilostazol	1 (0.8%)
-Aspirin + Clopidogrel	120 (99.2%)
ARU	445.4 ± 67.7
PRU	177.4 ± 69.5
Post-treatment DAPT	
-Aspirin + Cilostazol	1 (0.8%)
-Aspirin + Clopidogrel	99 (81.8%)
-Aspirin + Ticlopidine	21 (17.4%)
Device	
-Surpass Flow Diverter, Evolve	67 (55.4%)
-Surpass Flow Diverter, Streamline	31 (25.6%)
-Pipeline Embolization Device, Flex	22 (18.2%)
-Derivo Embolization Device	1 (0.8%)
Additional coil	3 (2.5%)
Number of fow diverters used	
-1	116 (95.9%)
-2	5 (4.1%)
Balloon angioplasty	40 (33.1%)
Procedure time (min)	91.1 ± 44.9
Angiographic follow-up (month)	26.0 ± 19.0
Clinical follow-up (month)	29.5 ± 22.2

ICA, internal carotid artery; ACA, anterior cerebral artery; MCA, middle cerebral artery; PCA, posterior cerebral artery; BA, basilar artery; VA, vertebral artery; mRS, modified Rankin Scale score; DAPT, dual antiplatelet therapy; ARU, aspirin resistance unit; PRU, plavix resistance unit.

**Table 2:** Clinical outcomes of the study subjects.

Complete aneurysm occlusion *	88 (72.7%)
Safety outcomes	11 (9.1%)
Hemorrhagic stroke	4 (3.3%)
Delayed RIPH	2 (1.7%)
Delayed rupture, SAH	2 (1.6%)
Major ischemic stroke ^†^	4 (3.3%)
Downstream embolic infarction	1 (0.8%)
Covered perforator territory infarction	1 (0.8%)
Stent thrombosis	2 (1.7%)
Stent thrombosis without infarction	2 (1.7%)
Sudden death, unknown cause	1 (0.8%)
Aneurysm enlargement ^‡^	14 (11.6%)
Unfavorable functional outcome ^§^	5 (4.1%)

RIPH, remote intraparenchymal hemorrhage; SAH subarachnoid hemorrhage; * Complete aneurysm occlusion was defined as the angiographic occlusion of the target aneurysm at the end of follow-up, without significant (>50%) parent artery stenosis, major adverse events, or the need for additional treatment; ^†^ Major ischemic stroke was defined as an increase of ≥4 points in the National Institutes of Health Stroke Scale score; ^‡^ Aneurysm enlargement was defined as a follow-up aneurysm volume exceeding 125% of the initial aneurysm volume; ^§^ Unfavorable functional outcomes defined as a modified Rankin Scale score of 3–6 at the last clinical follow-up.

**Table 3:** Logistic regression analysis for complete aneurysm occlusion after flow diversion.

Complete Aneurysm Occlusion *						
	Univariate Analysis			Multivariate Analysis		
	*p* Value	OR	95% CI	*p* Value	OR	95% CI
Age	0.311	0.98	0.95–1.01			
Male	0.552	0.77	0.34–1.83			
Posterior circulation	0.158	0.54	0.23–1.28	0.584	0.71	0.21–2.51
Non-saccular type	0.048	0.44	0.19–0.99	0.663	1.32	0.39–4.74
Aneurysm diameter	<0.001	0.89	0.83–0.95	0.009	0.89	0.82–0.97
Neck diameter	0.001	0.85	0.77–0.94	0.415	0.94	0.81–1.08
Incorporated branch	0.002	0.25	0.11–0.60	0.003	0.22	0.08–0.59
Number of flow diverters used	0.52	0.55	0.09–4.30			
**Safety Outcomes ^†^**						
	Univariate analysis			Multivariate analysis		
	*p* value	OR	95% CI	*p* value	OR	95% CI
Age	0.555	1.02	0.97–1.07			
Male	0.759	1.22	0.30–4.33			
Posterior circulation	0.850	0.88	0.18–3.24			
Non-saccular type	0.220	2.18	0.62–8.02			
Aneurysm diameter	<0.001	1.20	1.09–1.34	0.001	1.21	1.09–1.37
Neck diameter	0.068	1.10	0.99–1.23	0.702	0.98	0.85–1.11
Incorporated branch	0.483	0.57	0.08–2.35			
Number of flow diverters used	0.403	2.65	0.13–20.22			
**Hemorrhagic and Major Ischemic Stroke**						
	Univariate analysis			Multivariate analysis		
	*p* value	OR	95% CI	*p* value	OR	95% CI
Age	0.812	1.01	0.95–1.07			
Male	0.652	0.68	0.10–3.14			
Posterior circulation	0.293	0.32	0.02–1.89			
Non-saccular type	0.443	1.76	0.40–7.79			
Aneurysm diameter	0.003	1.18	1.06–1.33	0.003	1.18	1.06–1.33
Neck diameter	0.477	1.05	0.90–1.18			
Incorporated branch	0.881	0.88	0.12–4.07			
Number of flow diverters used	0.251	3.89	0.19–31.23			

OR, odds ratio; CI, confidence interval; * Complete aneurysm occlusion was defined as the angiographic occlusion of the target aneurysm at the end of follow-up, without significant (>50%) parent artery stenosis, major adverse events, or the need for additional treatment; ^†^ Safety outcomes included hemorrhagic stroke, major ischemic stroke (defined as an increase of ≥4 points on the National Institutes of Health Stroke Scale), partial or complete stent thrombosis, and all-cause mortality.

**Table 4:** Cox proportional hazard analysis for complete aneurysm occlusion after flow diversion.

	Univariate Analysis			Multivariate Analysis		
	*p* Value	HR	95% CI	*p* Value	HR	95% CI
Age	0.720	1.00	0.98–1.01			
Male	0.375	0.81	0.52–1.28			
Posterior circulation	0.673	0.90	0.56–1.45			
Non-saccular type	0.177	0.73	0.47–1.15			
Aneurysm diameter	0.037	0.97	0.94–1.00			
Neck diameter	0.006	0.93	0.88–0.98	0.009	0.92	0.87–0.98
Incorporated branch	0.001	0.39	0.23–0.66	0.001	0.40	0.24–0.69
Multiple flow diverters use	0.686	0.79	0.25–2.25			

HR, hazards ratio; CI, confidence interval.

**Table 5:** Comparison of baseline characteristics, treatment details, and treatment outcomes between early and recent groups.

	Total	Early Group	Recent Group	*p* Value
	(*n* = 121)	(*n* = 47)	(*n* = 74)	
Age (year)	59.1 ± 13.0	58.7 ± 14.2	59.3 ± 12.2	0.803
Female	82 (67.8%)	23 (48.9%)	59 (79.7%)	0.001
Anterior circulation	85 (70.2%)	29 (61.7%)	56 (75.7%)	0.151
Location				0.058
-ICA	75 (62.0%)	22 (46.8%)	53 (71.6%)	
-ACA	4 (3.3%)	3 (6.4%)	1 (1.4%)	
-MCA	6 (5.0%)	4 (8.5%)	2 (2.7%)	
-PCA	1 (0.8%)	1 (2.1%)	0 (0.0%)	
-BA	6 (5.0%)	2 (4.3%)	4 (5.4%)	
-VA	29 (24.0%)	15 (31.9%)	14 (18.9%)	
Non-saccular type	45 (37.2%)	25 (53.2%)	20 (27.0%)	0.007
Aneurysm diameter (mm)	15.2 ± 7.0	19.3 ± 6.2	12.6 ± 6.3	<0.001
Neck diameter (mm)	8.9 ± 4.8	10.3 ± 5.3	7.9 ± 4.1	0.005
Incorporated branch	33 (27.3%)	17 (36.2%)	16 (21.6%)	0.123
Device				<0.001
-Surpass Flow Diverter, Streamline	31 (25.6%)	31 (66.0%)	0 (0.0%)	
-Pipeline Embolization Device, Flex	22 (18.2%)	13 (27.7%)	9 (12.2%)	
-Surpass Flow Diverter, Evolve	67 (55.4%)	3 (6.4%)	64 (86.5%)	
-Derivo Embolization Device	1 (0.8%)	0 (0.0%)	1 (1.4%)	
Balloon angioplasty	40 (33.1%)	22 (46.8%)	18 (24.3%)	0.018
Procedure time (min)	91.1 ± 44.9	113.4 ± 52.8	77.0 ± 32.2	<0.001
Complete aneurysm occlusion *	88 (72.7%)	29 (61.7%)	59 (79.7%)	0.050
Aneurysm enlargement ^†^	14 (11.6%)	12 (25.5%)	2 (2.7%)	<0.001
Safety outcomes ^‡^	11 (9.1%)	8 (17.0%)	3 (4.1%)	0.036
All stroke	8 (6.6%)	7 (14.9%)	1 (1.4%)	0.011
-Hemorrhagic stroke	4 (3.3%)	4 (8.5%)	0 (0.0%)	0.042
-Major ischemic stroke ^§^	4 (3.3%)	3 (6.4%)	1 (1.4%)	0.324

ICA, internal carotid artery; ACA, anterior cerebral artery; MCA, middle cerebral artery; PCA, posterior cerebral artery; BA, basilar artery; VA, vertebral artery; * Complete aneurysm occlusion was defined as the angiographic occlusion of the target aneurysm at the end of follow-up, without significant (>50%) parent artery stenosis, major adverse events, or the need for additional treatment; ^†^ Aneurysm enlargement was defined as a follow-up aneurysm volume exceeding 125% of the initial aneurysm volume; ^‡^ Safety outcomes included hemorrhagic stroke, major ischemic stroke (defined as an increase of ≥4 points on the National Institutes of Health Stroke Scale), partial or complete stent thrombosis, and all-cause mortality; ^§^ Major ischemic stroke was defined as an increase of ≥4 points in the National Institutes of Health Stroke Scale score.

## Data Availability

The data presented in this study are available on request from the corresponding author due to ethical restrictions. The data contain sensitive patient information, and sharing it publicly could compromise patient confidentiality and violate privacy laws. Therefore, we can only provide the data to researchers who submit a reasonable request and agree to comply with the ethical guidelines and privacy regulations applicable to this data.

## References

[1] Becske T., Kallmes D.F., Saatci I., McDougall C.G., Szikora I., Lanzino G., Moran C.J., Woo H.H., Lopes D.K., Berez A.L. (2013). Pipeline for Uncoilable or Failed Aneurysms: Results from a Multicenter Clinical Trial. Radiology.

[2] Chancellor B., Raz E., Shapiro M., Tanweer O., Nossek E., Riina H.A., Nelson P.K. (2020). Flow Diversion for Intracranial Aneurysm Treatment: Trials Involving Flow Diverters and Long-Term Outcomes. Neurosurgery.

[3] Brinjikji W., Murad M.H., Lanzino G., Cloft H.J., Kallmes D.F. (2013). Endovascular treatment of intracranial aneurysms with flow diverters: A meta-analysis. Stroke.

[4] Cagnazzo F., Lefevre P.H., Derraz I., Dargazanli C., Gascou G., di Carlo D.T., Perrini P., Ahmed R., Hak J.F., Riquelme C. (2020). Flow-Diversion Treatment for Unruptured Nonsaccular Intracranial Aneurysms of the Posterior and Distal Anterior Circulation: A Meta-Analysis. AJNR Am. J. Neuroradiol..

[5] Jee T.K., Yeon J.Y., Kim K.H., Kim J.S., Hong S.C., Jeon P. (2021). Treatment Outcomes After Single-Device Flow Diversion for Large or Giant Aneurysms. World Neurosurg..

[6] Salem M.M., Sweid A., Kuhn A.L., Dmytriw A.A., Gomez-Paz S., Maragkos G.A., Waqas M., Parra-Farinas C., Salehani A., Adeeb N. (2022). Repeat Flow Diversion for Cerebral Aneurysms Failing Prior Flow Diversion: Safety and Feasibility From Multicenter Experience. Stroke.

[7] Jee T.K., Yeon J.Y., Kim K.H., Kim J.S., Jeon P. (2024). Evaluation of the Significance of Persistent Remnant Filling and Enlargement After Flow Diversion for Intracranial Aneurysms. World Neurosurg..

[8] Bender M.T., Colby G.P., Lin L.M., Jiang B., Westbroek E.M., Xu R., Campos J.K., Huang J., Tamargo R.J., Coon A.L. (2018). Predictors of cerebral aneurysm persistence and occlusion after flow diversion: A single-institution series of 445 cases with angiographic follow-up. J. Neurosurg..

[9] Oishi H., Teranishi K., Yatomi K., Fujii T., Yamamoto M., Arai H. (2018). Flow Diverter Therapy Using a Pipeline Embolization Device for 100 Unruptured Large and Giant Internal Carotid Artery Aneurysms in a Single Center in a Japanese Population. Neurol. Med.-Chir..

[10] Madaelil T.P., Grossberg J.A., Howard B.M., Cawley C.M., Dion J., Nogueira R.G., Haussen D.C., Tong F.C. (2019). Aneurysm Remnants after Flow Diversion: Clinical and Angiographic Outcomes. AJNR Am. J. Neuroradiol..

[11] Kallmes D.F., Brinjikji W., Boccardi E., Ciceri E., Diaz O., Tawk R., Woo H., Jabbour P., Albuquerque F., Chapot R. (2016). Aneurysm Study of Pipeline in an Observational Registry (ASPIRe). Interv. Neurol..

[12] Liu J.M., Zhou Y., Li Y., Li T., Leng B., Zhang P., Liang G., Huang Q., Yang P.F., Shi H. (2018). Parent Artery Reconstruction for Large or Giant Cerebral Aneurysms Using the Tubridge Flow Diverter: A Multicenter, Randomized, Controlled Clinical Trial (PARAT). AJNR Am. J. Neuroradiol..

[13] Martinez-Galdamez M., Lamin S.M., Lagios K.G., Liebig T., Ciceri E.F., Chapot R., Stockx L., Chavda S., Kabbasch C., Farago G. (2019). Treatment of intracranial aneurysms using the pipeline flex embolization device with shield technology: Angiographic and safety outcomes at 1-year follow-up. J. Neurointerv. Surg..

[14] Jee T.K., Yeon J.Y., Kim K.H., Kim J.S., Hong S.C., Jeon P. (2022). Early clinical experience of using the Surpass Evolve flow diverter in the treatment of intracranial aneurysms. Neuroradiology.

[15] Bonney P.A., Connor M., Fujii T., Singh P., Koch M.J., Stapleton C.J., Mack W.J., Walcott B.P. (2020). Failure of Flow Diverter Therapy: Predictors and Management Strategies. Neurosurgery.

[16] Bae H.J., Park Y.K., Cho D.Y., Choi J.H., Kim B.S., Shin Y.S. (2021). Predictors of the Effects of Flow Diversion in Very Large and Giant Aneurysms. AJNR Am. J. Neuroradiol..

[17] Rouchaud A., Brinjikji W., Lanzino G., Cloft H.J., Kadirvel R., Kallmes D.F. (2016). Delayed hemorrhagic complications after flow diversion for intracranial aneurysms: A literature overview. Neuroradiology.

[18] Daou B., Atallah E., Chalouhi N., Starke R.M., Oliver J., Montano M., Jabbour P., Rosenwasser R.H., Tjoumakaris S.I. (2018). Aneurysms with persistent filling after failed treatment with the Pipeline embolization device. J. Neurosurg..

[19] Issa R., Al-Homedi Z., Syed D.H., Aziz W., Al-Omari B. (2022). Surpass Evolve Flow Diverter for the Treatment of Intracranial Aneurysm: A Systematic Review. Brain Sci..

[20] Acerbi F., Mazzapicchi E., Falco J., Vetrano I.G., Restelli F., Faragò G., La Corte E., Bonomo G., Bersano A., Canavero I. (2022). The Role of Bypass Surgery for the Management of Complex Intracranial Aneurysms in the Anterior Circulation in the Flow-Diverter Era: A Single-Center Series. Brain Sci..

